# Polymyxin B-direct hemoperfusion therapy contributes to oxygen delivery in septic patients

**DOI:** 10.1186/cc11744

**Published:** 2012-11-14

**Authors:** A Yaguchi, M Yokota, T Goto, M Takeda, R Moroi, T Harada, M Namiki

**Affiliations:** 1 Tokyo Women's Medical University, Tokyo, Japan

## Background

Since 1994, Polymyxin B-direct hemoperfusion (PMX-DHP) (Toraymyxin^®^; Toray Medical Co., Tokyo, Japan) has been approved as a therapy for patients with severe sepsis or septic shock due to Gram-negative infection in Japan. However, its efficacy and indication are still controversial issues. Recently, randomized controlled studies are ongoing in other countries. Our hypothesis is that PMX-DHP therapy may improve the hemodynamic status in septic patients.

## Methods

From July 1994 to June 2010, all adult patients treated with PMX-DHP and receiving a pulmonary arterial catheter (PAC) in our ICU were included in this retrospective observational study. Patients' clinical, microbiological and PAC data were collected from medical archives. PAC variables were compared between immediately before and immediately after PMX-DHP therapy. Values were expressed as mean ± SD. Data were analyzed by Wilcoxon signed-ranks test, chi-square test and Fisher's exact probability test. *P *< 0.05 was considered statistically significant.

## Results

Sixty-three patients (36 men, 27 women; age range 24 to 89 years (mean 63.4 ± 14.8)) were studied. The mortality rate was 30.2% at 28 days after PMX-DHP. The APACHE II score and SOFA score on the day of PMX-DHP therapy were 20.2 ± 14.8 and 7.3 ± 3.8, respectively. Mean arterial pressure (MAP; mmHg), cardiac index (CI; l/minute/m^2^), and oxygen delivery and consumption (DO_2 _and VO_2_; ml/minute) significantly improved after PMX-DHP therapy (77.5 ± 22.5 vs. 84.1 ± 23.4, *P *= 0.0013; 3.9 ± 1.5 vs. 4.3 ± 1.2, *P *= 0.0006; 951.6 ± 403.5 vs. 1,002.5 ± 394.0, *P *= 0.013; 233.5 ± 80.6 vs. 248.0 ± 84.1, *P *= 0.004, respectively). The systemic venous resistance index (SVRI) (dyn second m^2^/cm^5^) and mixed venous oxygen saturation (SvO_2_; %) were not statistically different before and after PMX-DHP therapy (1,573.6 ± 614.6 vs. 1,527.7 ± 575.5, *P *= 0.069; 71.9 ± 9.0 vs. 74.1 ± 7.5, *P *= 0.082). The inotropic score and P/F ratio improved after the therapy (9.9 ± 15.9 vs. 7.5 ± 12.5, *P *= 0.018; 269.8 ± 108.5 vs. 300.6 ± 133.4, *P *= 0.003).

## Conclusion

MAP, CI, DO_2_, VO_2_, inotropic score and P/F ratio improved after PMX-DHP therapy, while SVRI and SVO_2 _did not change. PMX-DHP could contribute to oxygen delivery due to improve hemodynamic status, while decreasing inotropic agents in septic patients.

